# MicroRNA Signaling in Embryo Development

**DOI:** 10.3390/biology6030034

**Published:** 2017-09-14

**Authors:** Nicole Gross, Jenna Kropp, Hasan Khatib

**Affiliations:** 1 Department of Animal Sciences, University of Wisconsin-Madison, Madison, WI 53706, USA; ngross2@wisc.edu; 2 Wisconsin National Primate Research Center, University of Wisconsin-Madison, Madison, WI 53715, USA; jkropp@wisc.edu

**Keywords:** miRNA, embryo, extracellular vesicle, exosome, oocyte, sperm, implantation, signaling

## Abstract

Expression of microRNAs (miRNAs) is essential for embryonic development and serves important roles in gametogenesis. miRNAs are secreted into the extracellular environment by the embryo during the preimplantation stage of development. Several cell types secrete miRNAs into biological fluids in the extracellular environment. These fluid-derived miRNAs have been shown to circulate the body. Stable transport is dependent on proper packaging of the miRNAs into extracellular vesicles (EVs), including exosomes. These vesicles, which also contain RNA, DNA and proteins, are on the forefront of research on cell-to-cell communication. Interestingly, EVs have been identified in many reproductive fluids, such as uterine fluid, where their miRNA content is proposed to serve as a mechanism of crosstalk between the mother and conceptus. Here, we review the role of miRNAs in molecular signaling and discuss their transport during early embryo development and implantation.

## 1. Introduction

Mechanisms of communication between the mother and conceptus must be achieved for a successful pregnancy to occur. This hinges on crosstalk between maternal cells and the developing embryo. Various signals have been investigated in depth for their roles in the establishment and maintenance of pregnancy. These signals include hormones, growth factors, cytokines, and proteases [1,2,3,4]. Recent studies indicate non-coding, single-stranded nucleic acids, stretching 21–25 nucleotides in length [5,6,7], called miRNAs, are included in extracellular packages exchanged between cells [8]. miRNAs serve as a molecular switch for translation by binding to complementary sequences of a target mRNA within an RNA-induced silencing complex. Following binding, a target mRNA may become degraded, downregulated or even upregulated [5,9,10,11,12]. Interestingly, miRNA levels fluctuate by developmental state [13,14,15], cell type [16], and transport mechanism [17,18,19]. They are present at the earliest stages of embryo development, and can therefore serve as a tool of communication between the preimplantation embryo and the mother. Biogenesis and common regulatory properties of miRNAs are discussed in detail in previous reviews [5,7,9,10,11]. Therefore, this review will focus on their roles in mammalian gametogenesis and in signaling in the context of preimplantation embryonic development and embryo–mother crosstalk, with an emphasis on modes of transfer for miRNAs.

## 2. miRNAs in Gametogenesis and Preimplantation Development

**Figure 1:**
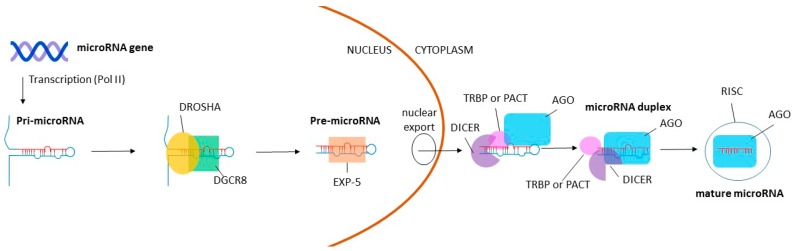
Basic pathway for miRNA biogenesis. Pri-miRNA is transcribed from a miRNA gene by RNA polymerase II (Pol II). DROSHA and DiGeorge syndrome critical region gene 8 (DGCR8) then bind together to form a microprocessor complex, which cleaves pri-miRNA to pre-miRNA. Pre-miRNA is exported from the nucleus to the cytoplasm by exportin-5 (EXP-5), where DICER converts pre-miRNA to mature miRNA along with TAR RNA-binding protein (TRBP) or PACT (also known as PRKRA) and Argonaute protein (AGO). DICER, TRBP or PACT, and AGO mediate duplex separation and loading of the mature miRNA into the RISC complex (RNA-induced silencing complex), whereby the miRNA can regulate gene expression.

The importance of miRNAs during gamete and early embryo development has been explored broadly. Several reviews outline current knowledge of miRNAs during oogenesis [20,21,22], spermatogenesis [23,24,25], and early embryogenesis [26,27,28]. The vitality of these miRNAs to both oogenic and spermatogenic pathways has been evaluated through in vivo knockout studies of several proteins involved in miRNA biogenesis, including DROSHA, DiGeorge syndrome critical region gene 8 (DGCR8), and DICER. During miRNA biogenesis, DROSHA and DGCR8 bind together, creating a microprocessor complex which facilitates cleavage of pri-miRNA to pre-miRNA. Following pre-miRNA export from the nucleus, DICER complexes with several proteins and cleaves pre-miRNAs into mature miRNA. The miRNA is then loaded into a RISC (RNA-induced silencing complex) whereby it can mediate mRNA expression [7] (Figure 1).

### 2.1. Oogenesis

Several studies have addressed whether miRNAs are an essential component for oogenesis. Initial evaluation of miRNA in the female reproductive system was assessed through a global knockdown of small RNA activity by targeting DICER. *Dicer1* is not specific to miRNAs because it has generalized functions in processing other small RNAs, namely siRNAs [7,29]. *Dicer1* conditional knockouts (cKOs) in mouse oocytes lead to severe effects such as infertility, abnormal chromosomal alignment, and disrupted spindle organization [30,31]. However, to specifically assess miRNA impact on oogenesis, additional regulators of the miRNA biogenesis pathways have been explored. Suh et al. [32] utilized a *ZP3* promoter-Cre recombinase system to eliminate *Dgcr8* from oocytes. When *Dgcr8* cKO mice were compared with controls, miRNA levels were dampened and fecundity was decreased. Regardless of a notable mature miRNA disparity, normally maturing oocytes were produced from *Dgcr8* cKO mice. When compared to *Dicer1* cKOs, *Dgcr8* cKO oocytes lacked the abnormal spindle formation seen in *Dicer1* cKO oocytes. Additionally, no differences in mRNA expression were noted between *Dgcr8* knockout and control oocytes [32]. The authors concluded that endo-siRNAs rather than miRNAs are essential for murine oogenesis to occur. They further suggested that a global halt on miRNA activity takes place in the mature oocyte and early zygote [32]. *Dgcr8* is primarily involved in processing canonical miRNAs [7], so it is still possible non-canonical miRNAs are capable of functioning independently of *Dgcr8*.

Importantly, these observations are made with healthy animals, and do not take into account the potential necessity of miRNAs in oogenesis which may occur under suboptimal or altered environmental conditions. Some changes of miRNA expression patterns which may be influenced by hormonal or age-related factors have already been observed. For example, miRNA expression is modified according to a follicle’s ovulatory status [33]. Furthermore, the oocytes of immature female mice (15–16 or 20–21 days after birth) and adult female mice have distinct miRNA composition [30]. *Dgcr8* knockouts have not yet been challenged with factors such as increased aging, undernutrition, heat stress, or disrupted paracrine signaling. However, it is possible that under conditions of environmental stress, canonical miRNAs may perform essential regulation of events such as follicle development and oocyte maturation. Therefore, future research into miRNA profiles in response to external changes or stressors on the reproductive tract is necessary.

Transgenerational effects of ovarian exposure to stressors have also been noted, although direct mechanisms have yet to be explained. In the female, it has been demonstrated that miRNAs may react to environmental exposures. Prenatal exposure of female sheep to hormones, such as androgens, triggered miRNA expression differences in the ovary of the female offspring from exposed ewes [34]. This effect carried through to the adult life of the offspring. Additionally, exposed female lambs developed a phenotype resembling polycystic ovarian syndrome (PCOS) [34]. The offspring of sows fed low protein diets during gestation also have altered miRNA expression in their ovaries, which is accompanied by various phenotypic differences, such as an increased number of secondary follicles and elevated 17β-estradiol [35]. Transgenerational effects indicate a potential for deeper roles of these miRNAs in developmental programming. A limited number of studies have investigated these lasting effects. Since both the maternal and paternal gametes fuse to form the embryo, it should be acknowledged that the sperm may also contribute to these effects.

### 2.2. Spermatogenesis

The sperm is the oocyte’s counterpart in fertilization. Similar models to those used for the investigation of miRNAs in oogenesis have been employed for the analysis of spermatogenesis. It is well accepted that miRNA gene regulation is essential for spermatogenesis to occur [36,37]. miRNA content and activity in the testis changes as pre-pubertal mice mature [38,39,40] and as spermatogenesis progresses [38,39,41]. As a preliminary investigation of small RNAs in spermatogenesis, *Dicer1* knockouts in mice were generated to assess the effects on germ cell development. Indeed, the deletion of *Dicer1* in the testis of mice [42,43] led to altered expression of miRNAs. However, while *Dicer1* knockouts result in a depletion of miRNAs and endo-siRNAs, *Drosha* deletion does not hinder endo-siRNA processing in the testes [43]. Thus, *Drosha* knockout models were developed for comparison because the RNase III enzyme is more specific to processing miRNAs than *Dicer1* [7]. A side-by-side testes knockout experiment showed that *Dicer1* and *Drosha* were each essential for spermatogenesis, since their deletion caused infertility [43]. Although no direct targeting comparison was performed, analysis of miRNA and mRNA expression revealed many dysregulated miRNAs and mRNAs in both knockout models [43]. The overall necessity for miRNAs in spermatogenesis is established. However, it remains a challenge to functionally show the roles of specific miRNAs influential to defects seen in knockouts. This is intertwined with the nature of miRNAs, since the small molecules have greater than 400 target mRNAs per family and each mRNA can be targeted by multiple miRNAs [44]. Thus, the direct and indirect effects of miRNA experiments are challenging to interpret. However, the necessity of miRNAs for spermatogenesis lends insight to the biological impact that different miRNA markers may have on spermatozoa themselves, and potentially on the freshly fertilized zygote.

Changes in hormones, stress, and diet can lead to alterations of miRNA expression during spermatogenesis. For example, a combination of nine miRNAs were found dysregulated in the spermatozoa of male mice under chronic stress. The nine miRNAs were injected into zygotes, leading to offspring exhibiting similar stress phenotypes [45]. Likewise, miRNAs disrupted by diet have also been shown to affect offspring [46]. miR-19b was upregulated in the sperm of mice consuming Western-like diets (high-fat, high-sugar diets). When miR-19b was injected into normal zygotes, it induced glucose intolerance phenotypes similar to those observed in the mice fed a Western-like diet [46]. A high-fat diet fed to male mice resulted in dysregulation of 13 miRNAs in the male’s sperm, where their male offspring and grand-offspring exhibited similar phenotypes, but did not have the same dysregulated miRNAs in their sperm [47]. In another study, the exposure of pregnant females to the anti-androgenic reproductive toxicant vinclozolin led to transgenerational effects in the offspring [48]. In three generations of paternally-derived male mice, fertility was decreased, the number of PGCs was lower, and apoptosis of PGCs was increased. miR-23b and miR-21 were dysregulated in all generations, leading to alteration of a major regulatory pathway, *Lin28/let-7/Blimp1* [48]. Brieño-Enríquez et al. [49] have reviewed additional cases where exposure of primordial germ cells (PGCs) to endocrine disruptors disturbed miRNA expression patterns. It is important to understand alterations in paternal miRNA content due to environmental factors because these miRNAs can contribute to the developmental potential of the embryo [50].

### 2.3. Fertilization and Early Embryonic Development

Upon successful penetration of the oocyte, the sperm delivers a package containing a variety of RNA species [51]. Yuan et al. [50] produced zygotes from miRNA-depleted sperm (from *Drosha* cKO mice) through intracytoplasmic sperm injection. The zygotes resulted in embryos with reduced developmental potential, which was recovered through the injection of small RNA from wild-type sperm. Further, the study used small, non-coding RNA-Seq to show that 14 miRNAs were present in wild-type sperm and 2-pronuclei embryos, but not in oocytes. Thus, several miRNAs are paternally contributed to the embryo from sperm and can affect the developmental potential of resulting embryos. The effects of individual miRNAs delivered to the zygote by the sperm are relatively unknown. However, miR-34c is thought to initiate the first cleavage divisions in the mouse [52], and represents a distinctive example of the impact of a single paternally-derived miRNA on embryo development.

The zygote and mature oocyte have similar miRNA profiles, which may suggest that many zygotic miRNAs are maternally inherited [30]. However, miRNA profiles change immediately after the first division of the embryo, where 60% of miRNAs are downregulated in the two-cell stage, indicating that many maternal miRNA transcripts may be disposed of following the first division [30]. It is important to be cautious when evaluating the roles of maternally-derived miRNAs, because their direct roles in embryo development have yet to be shown. However, it seems maternal miRNAs may still hold a role in producing a robust embryo. Intriguingly, when *Drosha* cKO sperm are injected into *Drosha* cKO oocytes, developmental potential decreases, and cannot be recovered by either sperm small RNA or total sperm RNA from wild-type sperm [50]. When wild-type oocytes were injected with *Drosha* cKO sperm, the effects on embryo development were recoverable through injection of wild-type sperm small RNA or total RNA. Therefore, the authors attributed lack of embryo recovery to the oocyte cKO miRNAs which were disrupted [50].

Unlike the gametes which fuse to form it, the zygote has the ability to differentiate into any cell type in the body. To facilitate this cellular transition, extensive molecular remodeling must occur during fertilization and early embryo development. Following fertilization, the embryo gains control of its own growth through embryonic genome activation, whereby the embryo begins to produce unique transcripts, rather than relying on parental gamete transcripts [53]. Recent reviews suggest the shift is facilitated through the alteration of epigenetic marks and potentially by the activity level of small RNAs [28,54].

Following fertilization of the murine metaphase II oocyte, the milieu of small RNAs in the embryo transitions from predominantly siRNAs and piRNAs at the zygotic stage to predominantly miRNAs by the blastocyst stage [55]. The regulation of this switch from the zygote to the blastocyst stage has been evaluated through the profiling of genes including *Drosha*, *Dgcr8*, *Exportin 5*, *Dicer*, *Ago1*, *Ago2*, *Ago3*, *Ago4* and *Ago5* [56]. These genes code for many of the biosynthetic proteins involved in processing miRNAs through canonical pathways. Interestingly, these genes are mainly downregulated following the zygote stage, with the exception that *Ago1*, *Ago3* and *Ago4* expression levels are increased after transition to the two-cell embryo, and *Ago2* is increased in four and eight-cell stage embryos [56]. The authors suggested that embryos therefore have reduced capacity for processing miRNAs as they progress from zygote to blastocyst [56]. However, they also found that mature miRNAs are bound in duplexes to mRNAs at the blastocyst stage, allowing for the embryo to reserve previously processed active miRNAs for functional use in the cells without the need for canonical processing [56]. Interestingly, another study which profiled the miRNAs of mouse oocytes and embryos from the one- to eight-cell stage through deep sequencing, found elevated 3’ mono- and oligoadenlyation, a modification which protects miRNA from degradation, in early zygote and two-cell embryos. This suggests that miRNA function is repressed during the oocyte to two-cell stage, and protected miRNAs may be re-activated following cleavage [57]. Thus, it is important to evaluate the availability and activity of miRNAs when assessing functional roles for miRNAs in the embryo.

**Figure 2:**
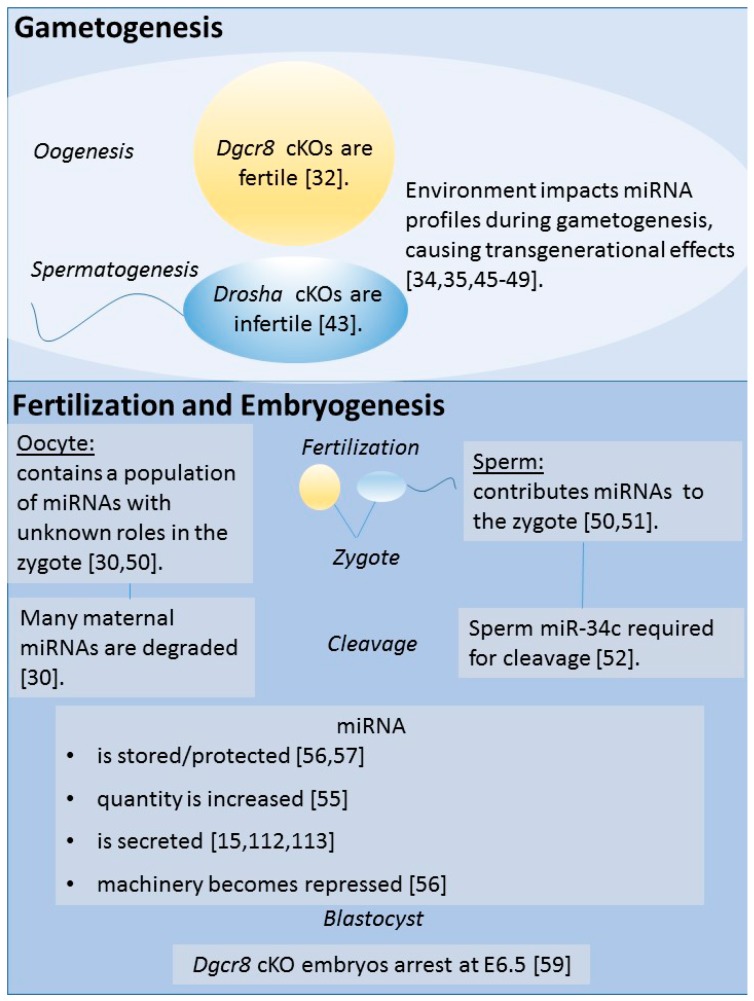
Links of miRNA to gametogenesis, fertilization, and embryogenesis. Knockout models have been developed to assess necessity of miRNAs in oogenesis and spermatogenesis. DGCR8 and DROSHA are both representative of protein knockouts specific to the microprocessor complex which cleaves pri-miRNA to pre-miRNA. Interestingly, females are fertile when this complex is disrupted, but males are infertile. Importantly, these models do not represent environmental impact which can occur to alter miRNA profiles. At fertilization, both the sperm and the oocyte contribute miRNA. However, the oocyte’s miRNA is rapidly degraded, so its use remains undetermined. Alternatively, it has been shown that a sperm RNA, miR-34c, is required for the first cleavage division following fertilization. In remaining divisions, control over miRNA availability is dynamic in the embryo.

Several knockout studies have shown that small RNAs, including miRNAs, are necessary for embryonic development. Both *Dicer1* and of *Dgcr8* knockouts in mice are embryonically lethal [58,59,60]. Embryos devoid of *Dicer1* are non-viable and have decreased numbers of stem cells [58]. *Dicer1* knockout lethality was demonstrated through heterozygous intercrosses, with no homozygous *Dicer1—*null offspring born of the 62 pups produced [58]. Moreover, *Dgcr8* knockouts have been generated to assess the viability of embryos lacking miRNA machinery. A study which generated zygotic *Dgcr8* mouse knockouts found that development arrested at E6.5 in mouse embryos [59]. Figure 2 summarizes links between miRNAs and gametogenesis, fertilization, and embryogenesis.

Additionally, some tissue-specific knockouts can cause lethality in later stages of embryo development. For instance, cKO of the crucial miRNA-specific processor *Dgcr8* from vascular smooth muscle cells in mouse embryos is embryonically lethal between E12.5 and E13.5, yielding no viable homozygous offspring from 78 pups born [60]. Therefore, researchers must be careful when developing tissue-specific knockouts for miRNA research in embryos. Further, the roles of specific miRNAs in embryonic progression must be evaluated, in addition to their activity under various conditions.

Dysregulation of miRNAs in embryos can potentially impact cell differentiation, implantation or maintenance of early pregnancy. Though few studies have performed deep investigation of these roles, the importance of miRNAs in successful embryonic development has been illustrated. For instance, murine embryos fertilized in vitro compared with those fertilized in vivo exhibit consistent downregulation of miR-199-5P at both the blastocyst (E3.5) and the epiblast (E7.5) stages of mouse embryo development. Through inhibitor (induced downregulation) and mimic (induced upregulation) experiments authors have demonstrated that the dysregulated miR-199-5P induced high glycolytic rates in blastocysts. These differences led to lineage misallocation and increased fetal losses, offering an explanation for differences seen between the success of in-vitro-fertilized versus in-vivo-fertilized embryos [61]. During these early time points of development, an embryo’s environment and establishment of communication with the mother is imperative. However, the roles of miRNAs in embryo–mother communication have not yet been fully uncovered.

## 3. Extracellular miRNAs

miRNAs are essential to many cellular process within a cell, and can be transferred between cells to serve as a mode of cell–cell communication [8]. Their structure and size, in addition to various transport mechanisms, allow them to exist stably within various biological fluids [62,63]. miRNAs existing in fluids can survive unruly conditions, including low pH, boiling and freezing [64]. Most bodily fluids including saliva [65], urine [66], plasma [67,68], serum [68], blood, tears, amniotic fluid, colostrum, breast milk, bronchial lavage, cerebrospinal fluid, peritoneal fluid, pleural fluid, seminal fluid, follicular fluid [69,70] and uterine fluid [71] contain miRNAs [72]. The presence of miRNAs in reproductive fluids such as follicular fluid [69,70], uterine fluid [71] (reviewed by Floris et al. [73]), and seminal fluid [72] demonstrates that miRNAs are present as circulating factors which could be transported along reproductive systems. Fluid-derived miRNAs could have roles in germ cell formation, preparation of maternal tissues for implantation, or in fetal–mother signaling during embryo development. Therefore, many of these reproductive fluids are under study for their ability to serve as early or non-invasive biomarkers of idiopathic infertility [74] and for their utility in early diagnosis of pregnancy complications [75,76]. The presence of miRNAs in fluids potentially allows miRNAs systemic access to distant cells in the body. For example, it is plausible, but currently unknown, that systemic miRNAs could signal to the pituitary gland to alter hormone regulation in the response to a reproductive event.

### 3.1. Modes of Extracellular miRNA Transport

miRNAs can be transported through biological fluids in a variety of ways. Extracellular miRNAs in fluids are transported by lipoproteins (both HDL and LDL) [77,78], bound to other proteins (Argonaute2 (AGO2) [17,18] and nucleophosmin 1 (NPM1) [79]) or are contained within EVs such as apoptotic bodies [80], microvesicles (MVs) [81], and exosome-like vesicles (reviewed by Raposo et al. [82] and Zampetaki et al. [83]). These modes of transportation protect the miRNAs from degradation and contribute to their stability in fluids [64]. Interestingly, cells secreting miRNAs have different miRNA profiles than their secretions indicate [84]. It has even been shown that some extracellular miRNAs have higher expression in EVs compared to the original cell from which they were secreted [8]. To understand this exchange, the mode of transport must be determined. miRNAs contained in EVs have been studied extensively (reviewed by Raposo et al. [82] and Cesi et al. [85]). The term EV is general and includes MVs (50–1000 nm in diameter), exosomes (40–100 nm in diameter), and apoptotic bodies (50–5000 nm in diameter) [86]. Common difficulties in the study of EVs are the consistency of their isolation [87] and their characterization [88]. For exosomes in particular, multiple methods are employed to verify their purity, including size determination and the presence of marker proteins (examples include CD63, TSG101, HSP90B1 and ACHE) [88]. Studies of exome biogenesis specify that exomes bud from endosomes (reviewed by Schorey et al. [89]). Since MVs overlap in size, exosomes thus cannot be completely distinguished from small MVs without disruption of endosomal activity. Therefore, it has been suggested by the International Society of Extracellular Vesicles to use the general abbreviation EV when referring to vesicles isolated from extracellular fluids [88]. To ensure compliance with these guidelines for defining EVs, we will use the abbreviation EV when referencing publications that use the term exosomes.

Research shows that EVs can transport a wide range of supplies from one cell type to another and are thought to be packaged selectively. For example, Squadrito et al. [90] used macrophages and endothelial cells to demonstrate that the sorting of miRNAs into EVs for heterotrophic cell communication is altered both by the presence of target transcripts and self-presence of the respectively sorted miRNA. Additionally, the isoforms of miRNAs represented in exosomes compared with the isoforms of parent cells differ [91]. The sequence of miRNAs can affect loading, since an EXOmotif (GGAG) has been shown to control miRNA allocation to exosomes [92]. These tiny spheres are able to carry proteins, mRNAs, DNA, and miRNAs [93], which are recorded in the online database ExoCarta (www.exocarta.org) [94]. Thus, their transport to recipient cells could represent a pre-designed cocktail of supplies to aid in communication and reformation with these cells. Similar to miRNAs, EVs have been found within many of the reproductive fluids which serve as complex sources of nutrients for gametes. These vesicles are named for their locations. Prostatosomes or prostasomes exist in seminal fluid [95,96], epididymsomes are in the epididymis [97,98], oviductosomes are in the oviduct, and uterosomes are in the uterus [99,100]. EV trafficking in the ovary and the epididymis has been evaluated and reviewed previously [70,74,101,102,103]. The transfer of information through EVs from sperm to sperm, male or female somatic cells to sperm, or sperm to oocyte has not yet been shown. However, if EVs can transfer, they may serve as a mode for sperm to communicate with each other, somatic cells they encounter, and the oocyte. Currently, EV profiles have been identified in many placental studies, and have been shown to vary based on disease state [104,105].

Other carriers of miRNAs such as lipoproteins, apoptotic bodies, and proteins should not be discounted for their potential roles in signaling. Notably, the proportion of cell-free miRNAs contained in EVs is controversial. It has been reported that the majority of miRNAs in serum and saliva are found in EVs [19]. However, Turchinovich et al. [17] assessed extracellular miRNAs in stable blood plasma and cell culture media, determining that most identified miRNAs were EV-free. Authors later found that these miRNAs are instead bound to AGO2 and suggested they are released through apoptosis. Additionally, authors determined that suites of miRNAs are separated by vehicle (protein vs. EV) [17]. Similar findings were also presented in another study [18]. Additionally, it is possible that RNA released by apoptotic vesicles can be translated in other cells [106], but the control of the cells over signaling through apoptotic vesicles is not well studied. Such apoptotic signaling would be in the case of a single-time release of supplies, upon cellular death. As such, EVs have been more heavily investigated. However, apoptotic cells release signals to indicate they should be phagocytized [107,108]. These ‘eat me’ signals could impact other signaling factors because phagocytes can release signals upon internalizing apoptotic bodies, such as vascular endothelial growth factor [109].

### 3.2. miRNAs in Biological Fluids

Regardless of the transport mechanism, circulating miRNAs are relatively stable [17]. The transport of miRNAs within fluid makes them particularly accessible, with the potential to avoid invasive procedures to assess the health of a subject. Thus, they are strong candidates as biomarkers for disease states [110]. It is therefore important to understand the natural state of these miRNAs in healthy subjects [111]. Their roles in biological regulation at times of dynamic change in the body such as pregnancy or disease can then be better understood. The type of fluid which contains them may give an indication of the extent to which given miRNAs can travel. For example, blood circulates systemically, whereas uterine fluid remains relatively localized. In a reproductive context, fluids such as semen are transferred from one individual to another. It has already been shown that follicles with mature oocytes differ in the follicular fluid miRNA milieu from those follicles containing immature oocytes. These miRNAs can be taken up by follicular cells in culture when they are supplemented with the follicular fluid [70]. This is one example of miRNA’s role in signaling through fluid to facilitate reproductive maturation.

In an in vitro context, fluids such as culture media from both mammalian cell cultures [16,17,79] and embryo culture [112,113] also contain miRNAs. These miRNAs can originate from components of the medium itself [17,114], such as fetal calf serum [68], fetal bovine serum [68], horse serum [68] and can be secreted by cells [16,17,79] and embryos [15,112,113]. miRNAs in media from embryos or culture cells can be linked to traits such as cell type [16,115] and development [13,14,15]. Thus, media-derived miRNA offers an opportunity to assess a functional read out of the biological mechanisms within embryos and cells, which allows for non-invasive biomarker development. Several studies have taken advantage of these models for the investigation of preimplantation signaling. For example, the reactions of maternally-derived culture cells to mimics of embryo-secreted miRNAs have been shown [116,117]. However, the impact of specific miRNAs needs the further support of in vivo studies. Additionally, the mechanisms of miRNA transport have yet to be fully investigated.

## 4. Cellular Communication

Three general forms of cellular communication define successful signaling. Crosstalk can be initiated through direct cell–cell contact (autocrine), through short-range secreted signals (paracrine), or through long-range secreted signals (endocrine). Through the secretory pathways of cellular communication, molecules such as hormones, growth factors and cytokines are exported from host cells and subsequently transported to target cells, where they recognize and bind to specific receptors. The action of these molecules can lead to alterations in cellular metabolism, proliferation, function, potency and viability.

**Figure 3:**
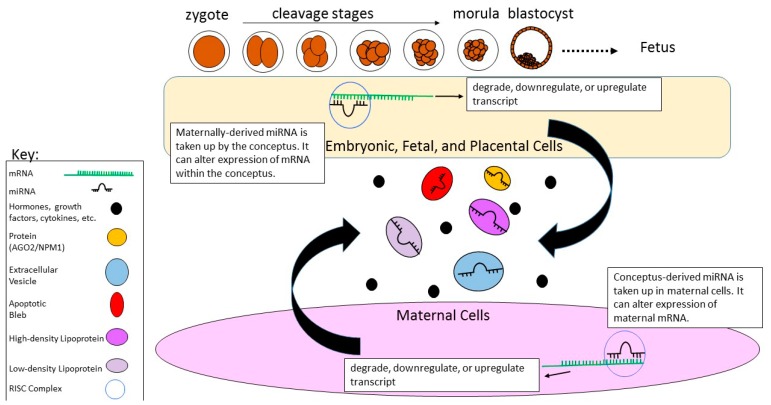
Schematic representation of miRNA crosstalk between a conceptus and mother. miRNAs have the potential to be transferred between maternal cells (represented as endometrium) and conceptus through various types of vehicles. These vehicles can be proteins (Argonaute 2 (AGO2) and Nucleophosmin 1 (NPM1)), extracellular vesicles, apoptotic blebs, high-density lipoproteins, and low-density lipoproteins. Upon delivery to either the mother or conceptus, the miRNA becomes bound to a RNA-induced silencing complex (RISC) and pairs to a complementary target mRNA. The target mRNA is then degraded, downregulated, or upregulated. This crosstalk can occur through direct cell–cell contact or miRNAs can be circulated and transferred in fluids, such as uterine fluid.

miRNAs can fulfill autocrine, paracrine, and endocrine signaling roles. They are reported to pass to other cells through structures such as gap junctions [118,119] as well as intercellular bridges and synapses [84,119]. Additionally, transcytosis has been suggested as a mechanism for miRNA transfer, thus allowing them to slip through biological barriers [120]. Vehicles of miRNAs, EVs, can enter cells through endocytosis, pinocytosis, fusion or by binding to surface receptors [85,121]. Regardless of the proportion of circulating miRNAs contained within EVs, miRNAs serve as cell–cell communication molecules. miRNAs have demonstrated the capacity to be passed from one cell to another, affecting translation within other cell types [8,122]. Valadi et al. [8] elegantly illustrated that EVs are capable of delivering both miRNAs and mRNAs to recipient cells, where the encapsulated mRNAs could be translated within the recipient cell upon delivery [8]. Likewise, Thomou et al. [122] showed that miRNAs can be transported through the bloodstream from adipose tissue to the liver in the mouse, thus demonstrating that EVs can be transported from one tissue type to another [122]. It has been suggested that miRNAs are the oldest evolutionary form of hormones [123]. Indeed, nuclear-receptor-mediated signaling, which is integral to hormonal activity, regulates miRNA expression (reviewed by Yang et al. [124]). Few in vivo studies have described a functional role of EV miRNA within a recipient cell or tissue. However, the ability for both neighboring and distant cells to affect each other through this transport has the potential to serve as a form of embryo–mother communication (Figure 3).

## 5. Conceptus–Mother Crosstalk

The maternal environment must recognize the embryo to initiate a pregnancy. This requires substantial regulation of the mother’s immune system, blood flow, and of the endometrium to initiate decidualization [125]. The changes to the embryo are also complex. Nutrients must be supplied to the embryo, and alternatively by-products produced by the embryo must be removed through the mother’s system. These processes are necessary to support the fertilized zygote through a series of cleavage events. Finally, cellular differentiation occurs, and a distinct inner cell mass (ICM) and an outer trophectoderm (TE) form at the blastocyst stage prior to implantation. The ICM must retain pluripotency to become the fetus, and the TE will differentiate into extraembryonic tissues to form the placenta [126,127]. Since both the embryo and the mother are undergoing constant changes to progress through the stages of pregnancy, substantial crosstalk is required.

In the human, considerable crosstalk occurs during establishment of immune tolerance to pregnancy, modulation of extravillous trophoblast invasion, and spiral artery remodeling [125]. Overall, there are several known mechanisms of conceptus–mother crosstalk [1,125,128,129,130]. Signals exchanged include cytokines, chemokines, proteases, antiproteases, and growth factors [1,2,3,4,125,128,129,130]. The interactions between these signals are difficult to attribute to a single pathway and the preparation of the uterus for implantation relative to these signals is still a mystery [131].

Circulating hormones are stimulated by embryo interaction with the endometrium, where a variety of local signals are present near implantation [125,128,129,130]. However, mechanisms for initiation of hormonal signals by the embryo are not well defined. It is possible that miRNAs serve a role to regulate a window of receptivity for implantation by altering levels of these hormones. Indeed, many ovarian hormones involved in establishing and maintaining pregnancy, including estrogen, human chorionic gonadotropin (hCG) and progesterone [2] have receptors which interact with miRNAs at the transcriptional and translational level, such as the estrogen receptor, or progesterone receptor (reviewed by Yang et al. [124]). For example, aromatase in granulosa cells is targeted by miR-378, which binds to two regions of the aromatase transcript leading to the downregulation of estradiol and aromatase synthesis [132]. Similarly, miRNA profiles are affected by hormonal levels in the circulation, including hCG, which is essential for recognition of pregnancy in the human [133]. Treatment of mice with hCG has been shown to increase miR-132 and miR-212 [134]. The regulation of ovarian steroid production by miRNAs is further reviewed by Li et al. [135]. However, hormonal shifts are not the sole requirement for receptivity. Control of the maternal immune response is highly important to establishing and maintaining pregnancy [125]. For example, seminal fluid expelled during coitus will initiate the regulation of maternal immune responses during pregnancy [136]. miRNAs are also involved in immune cell development in endometrial lymphocytes and have been investigated for their roles in the regulation of histocompatibility antigens, a feature which is limited to trophoblasts of the placenta [137].

The conception of multiple embryos also presents a unique signaling environment. Although the conception of twins in humans is not typical, many model organisms, including mice, pigs, and sheep typically maintain multiple fetuses upon conception. This has long been known as a challenge for the design of research studies [138]. Additionally, most embryo culture systems, including those for human in vitro production, utilize group culture systems. Therefore, it is important to acknowledge inter-embryo crosstalk. Cohorts of in vitro embryos can exchange a variety of signals, including proteins, lipids, hyaluronic acid, and amines [139]. It was also recently shown that porcine parthenogenic embryos can exchange EVs with cloned embryos [140]. As such, researchers must be mindful of possible differences in communication for mothers with multiple fetuses compared with those maintaining a singleton pregnancy. Comparisons of miRNAs between species must therefore be evaluated with caution, particularly for experiments where interactions of a single embryo cannot be discerned.

## 6. Recent Developments in Conceptus–Mother Communication Through miRNAs

In vitro culture systems offer a platform to assess both embryos and their secretions across developmental stages. Preimplantation of in-vitro-produced embryos that are morphologically similar near the time of transfer differ greatly in miRNA expression in relation to parental fertility status [141], sex of the embryo [142], chromosomal make-up [142], and developmental potential [15,112]. Similarly to other cell types, embryos also secrete miRNAs into human [112,113] and bovine [15,112] in vitro culture media. A survey of bovine blastocyst miRNA expression and corresponding in vitro culture media revealed that miRNAs are preferentially secreted from the embryo, as miR-25 was expressed in bovine blastocysts and corresponding culture media compared to miR-302c, which was solely detected in blastocysts [112]. Preferential or differential secretion of miRNAs was also observed between embryos of differing genetic composition [113,117] and developmental potential [15,113,115,116]. Rosenbluth et al. [113] reported that miRNA expression levels of miR-191, miR-372 and miR-645 were increased in the media of failed IVF cycles compared to the media of embryos that resulted in live birth. Similarly, Capalbo et al. [115] identified increased concentrations of miR-20a and miR-30c in the culture media of euploid-implanted blastocysts compared to unimplanted blastocysts. miRNA profiling of culture media and trophectoderm cells revealed similarities in miRNA expression [115], which suggests that the extracellular miRNAs are plausibly secreted by the trophectoderm. Secretion of miRNA within EVs has been identified in conditioned culture media derived from a trophoblast cell [143] and primary human trophoblasts [144]. These studies demonstrate that miRNAs are selectively secreted by embryos and trophoblasts into the extracellular environment, and thus could have a functional role at the maternal–fetal interface.

### 6.1. Embryo-Secreted miRNAs

Implantation is a complex process that relies on the coordinated expression and secretion of molecular cues in the uterine microenvironment including growth factors and integrin binding [145,146,147]. These processes are also plausibly mediated by secreted miRNA from the developing embryo present in the uterine fluid. Indeed, in vitro cell culture experiments have shown that miRNAs supplemented to endometrial epithelial cell culture medium modulate the gene expression of cultured cells [116,117]. Gross et al. [117] reported increased expression of miR-22 and miR-320a in bovine endometrial epithelial cells following the supplementation of synthetic miRNA mimics of miR-22 and miR-320a to the culture medium. Interestingly, increased expression of the progesterone receptor transcript, a target mRNA of both miR-22 and miR-320a, was observed, suggesting that miRNAs are able to positively upregulate expression through both indirect or direct mechanisms, as previously described [9,11,148]. The role of extracellular miRNA on maternal epithelial gene expression was also observed in human endometrial epithelial cell cultures (HEECs). Supplementation of fluorescently tagged miR-661, a miRNA present in non-implanted blastocyst conditioned medium, to HEECs demonstrated that the cells internalized extracellular miR-661, as confirmed by presence of immunofluorescence in the cytoplasm [116]. Moreover, extracellular miR-661 reduced trophoblast spheroid adhesion to HEECS establishing a functional role of extracellular miRNAs at the maternal–fetal interface.

Internalization of embryo-derived EVs by uterine cells in vivo revealed modulation of maternal gene expression. A survey of miRNA expression in uterine fluid consistently identified the presence of miR-16 and miR-125, embryo-derived miRNAs, in the uterine fluid of pregnant sows [149]. The predicted mRNA targets of these miRNAs have roles in pathways regulating pregnancy-related growth factors, receptors and cytokines, suggestive of an influence on the molecular mechanisms of implantation. Mechanistically, it has been shown that embryo-derived vesicles that may contain miRNA can be internalized by uterine cells. Burns et al. [150] infused fluorescently labeled embryo-derived EVs into the uterine horn near the time of implantation (d8-14 of cyclic ewes) and observed fluorescence in the cytoplasm of the uterine luminal epithelium and glandular epithelium. These studies establish that extracellular miRNAs originating from the embryo are internalized by uterine cells and modulate maternal gene expression, suggestive of a functional signaling role between the blastocyst and maternal endometrium near the time of implantation.

### 6.2. Extracellular miRNAs of Maternal Origin

Maternal extracellular miRNAs also serve as signaling molecules to the early blastocyst. External miRNAs present in embryo culture media are internalized by preimplantation stage embryos and functionally impact development. For example, supplementation of a miR-24 mimic to embryo culture media increased the expression of miR-24 in bovine blastocyst stage embryos 44-fold, and also greatly reduced embryonic development compared to control embryos [15]. Similarly, internalization of EV-associated and free miR-30d was observed in mouse embryos, resulting in the overexpression of genes involved in embryo adhesion including Itgb3, Itga7 and Cdh5 [151]. Furthermore, upon supplementation of murine embryos with miR-30d, embryo adhesion was improved, suggesting that external miRNAs have a functional role in mediating the processes of implantation. Profiling of miRNA expression in endometrial epithelial cell culture medium [151,152] and uterine fluid [71,151] revealed that maternally-derived miRNAs are present within EVs in the uterine microenvironment. Despite visualization of the internalization of maternally-derived vesicles, the mechanism(s) by which external miRNAs are taken up by the embryo is unknown. Uptake of both free-form synthetic miRNA mimics as well as vesicle-enclosed miRNAs has been shown in vitro, however, the internalization of free or protein-bound miRNA in an in vivo context is unreported.

Implantation is a complex process mediated by complex interactions and molecular cues, in which miRNA crosstalk between the conceptus and uterus has recently been investigated. Several questions remain unanswered as far as the nature and mode of action of miRNA at the maternal–fetal interface. The profiling of EVs in the uterine microenvironment has revealed a diverse miRNA milieu, however, the protein-bound or EV-free fractions remain uncharacterized. The labeling of EVs as performed by Burns et al. [150] showed that EVs can be internalized by both the conceptus and uterine endometrium. However, the questions of which miRNAs are internalized and how EVs and miRNAs are internalized remain unanswered. Internalized miRNAs then modulate target gene expression, although there are a vast number of genes and pathways that could be targeted by a single miRNA, posing a challenge to studying the specific effects of single and populations of miRNA. Moreover, in vitro experiments are limiting as they cannot encompass all interactions that are present in vivo during embryo development and implantation, thus optimizing in vitro conditions to emulate the in vivo environment should be a consideration for future experiments.

Moving forward, extracellular miRNAs could also serve as biomarkers of embryo development and the overall health status of the pregnancy. The differential expression of extracellular miRNAs in embryo culture media between embryos of differing developmental competency has already been documented [15,113] and could be used as a noninvasive method to select embryos prior to embryo transfer. Likewise, placenta-specific miRNAs are secreted into maternal plasma [143,153,154], where certain miRNAs have been associated with the health of the pregnancy [75,155,156,157,158]. Therefore, the extracellular miRNA milieu should be further characterized for not only the molecular roles in signaling but also as non-invasive biomarkers of embryo development and pregnancy.

## 7. Future Directions of miRNAs in Signaling in Reproduction

Overall, miRNAs seem to play an important role in reproduction and their function is fitting for early embryo development, where post-transcriptional regulation by miRNAs is an absolute necessity for embryo survival. Evaluating the roles of extracellular miRNAs could improve our understanding of communication during conception. Currently, there is a dearth of knowledge of embryo adaptation to the changing environment, modes of hormonal regulation, and fetal–maternal communication. However, many of the potential roles of miRNAs have yet to be understood in a functional context or harnessed in a biologically relevant manner. Although the presence of miRNAs in biological fluids and medium has been identified, the sources from which these miRNAs stem and the actual activity levels of the miRNAs found are relatively unexplored in reproductive processes. It is important to distinguish between miRNA activity versus presence. To achieve contextual knowledge of the roles these miRNAs play, it is important to develop proper models for the functional interrogation of miRNA roles following initial profiling.

Several technologies can be utilized to enable our understanding of these roles. CRISPR-Cas9 platforms offer an advantage to developing new knockout models, whereby the biogenesis and/or activity of miRNAs can be more specifically altered. Additionally, models of long-term cell culture can provide an advantage and a new window into understanding development. It is now possible to culture human embryos longer than ever before, up to day 13 [159,160]. Though miRNAs are shown to exist in the many stages of gamete development and across embryonic stages, most are functionally undefined in the context of their cellular environment both in natural and diseased states. Thus, along with a lengthened window of development, researchers should strive to understand basic cellular reactions in the context of miRNA production. Further, the new technology for single cell profiling can be used not only to assess the quality of gametes, but also to determine which cells are most active in terms of miRNA content and exchange. Profiling on the level of individual cells allows for the dissection of gametes to understand variation between stages of development, and competent versus incompetent gametes. In early development, the gametes contain miRNAs, which could be used by the early embryo. Gamete fusion itself is a unique form of cell-to-cell communication. Thus, single cell transcriptomic and epigenomic profiling could be used to evaluate these contributions. This technology could also be harnessed to evaluate the sources of secreted miRNAs from embryos. For example, it has been found that trophectoderm is a likely culprit for the miRNAs found in embryo culture media [115]. However, since it is believed the trophectoderm and inner cell mass exchange miRNAs, it would be intriguing to map this interaction through single-cell profiling. Similarly, comparison of miRNA exchange should be done at the site of embryo–mother communication.

The roles of miRNAs in the regulation of reproductive hormones deserves further investigation. Many hormones which show a strong interaction in the pituitary may be regulated on-location rather than at the site of reproduction. Thus, when evaluating miRNA that can interact with hormonal levels (which has been demonstrated for estrogens and androgens [124,132]) it will be crucial to evaluate other locations where those hormones are active in the body. It is already known miRNAs can elicit effects throughout the body [122], since they can be carried in EVs, so their destination or origin must be carefully interpreted. Additionally, since hormonal systems for the regulation of reproduction in animals are diverse, and they should be validated across species.

The modes through which miRNAs are secreted and travel must be better understood. However, there are a variety of challenges that come with the isolation of EV RNAs including purification, analysis and general characterization (reviewed by Mateescu et al. [161]). It is possible to track the uptake of EVs generally, but new techniques must be developed to increase the specificity of markers for EVs. Adding to this challenge, EVs are still hard to classify. While EVs are partially distinguished by the presence of specific proteins on their surface and their size [88], this does not fully explain their successful transfer from one remote tissue to another throughout a body, nor does it describe how they differ in content. EVs themselves have many destinations, but the way this occurs is unknown. These vesicles need to be characterized based on their eventual destination and their presence in specific types of biological fluids. They should be evaluated not only for their miRNA content, but also for the mRNAs, proteins, cytokines, or DNA they may contain.

It is important to remember that EVs are not the sole potential carriers of miRNAs in the two-way dialogue between an embryo and its mother. Proteins and EVs have been shown to contain different assortments of miRNAs [17] and could both be used as signaling vessels for differing purposes. The dimorphism in the cargo of these vessels likely extends to mRNA and protein content. Thus, macromolecules and other nucleic acids should be profiled in addition to miRNAs within these carriers. The distance which each of these vesicles can travel will affect the usage, but is unknown. Other vehicles of miRNA, such as LDL, HDL and proteins have been largely untouched in terms of their roles in communication during pregnancy. How the contents of these vesicles become partitioned to particular vesicles needs to be explored, as well as the percentage of miRNAs secreted by both the embryo and the mother which are contained in each type of vessel. Understanding the specific modes of transfer for miRNAs will aid the development of biomarkers because the mode of secretion not only helps to indicate cell state, but can also potentially indicate where the vessel’s cargo is going. In the future, it may also be possible to then specifically modify these vesicles and target disorders of pregnancy.

**Figure 4:**
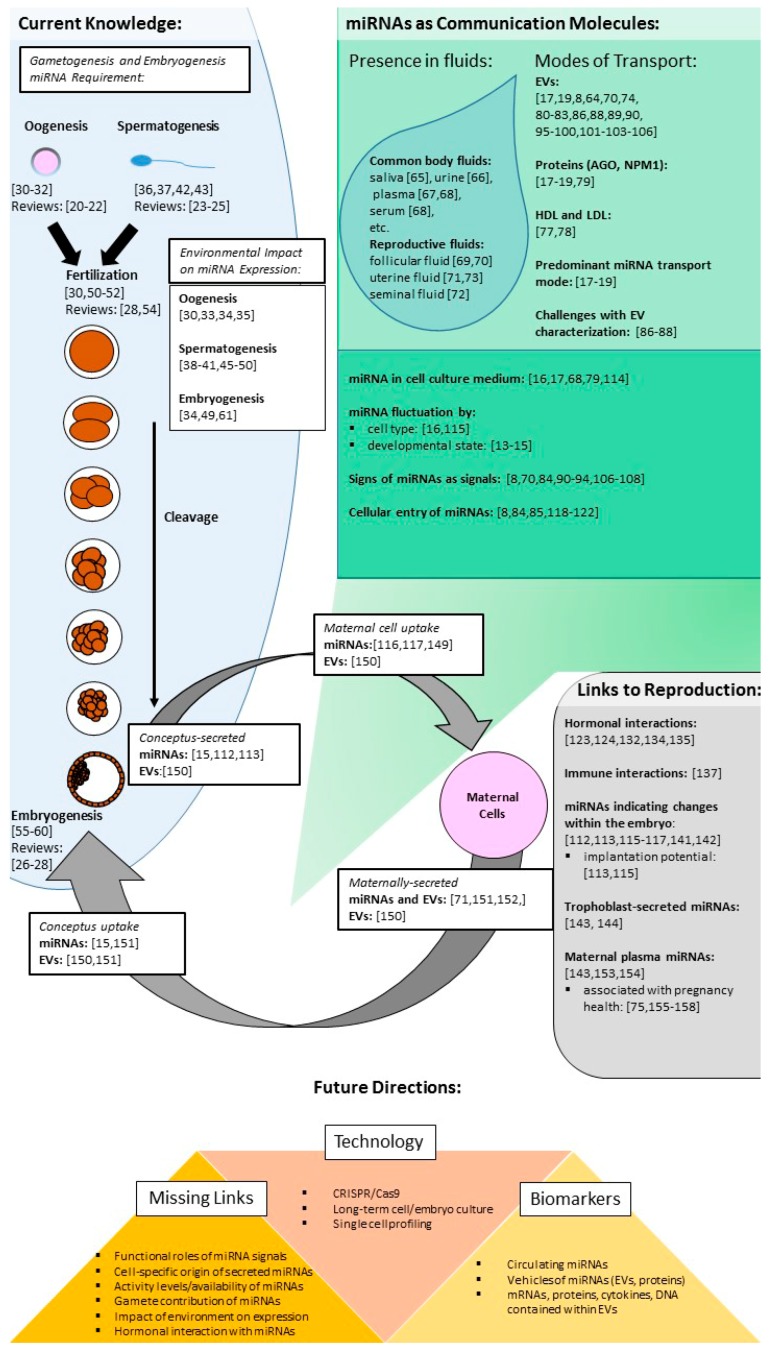
Summary of references on current knowledge of miRNAs in embryo development and signaling. A general schematic of references found throughout this article which outlines the links between miRNAs, embryo development, and signaling. Terms: extracellular vesicles (EVs), high-density lipoprotein (HDL), low-density lipoprotein (LDL), Argonaute (AGO) and nucleophosmin 1 (NPM1), microRNA(miRNA).

MiRNAs have been evaluated for their necessity in oogenesis and spermatogenesis. Thus far, it has been determined miRNAs are vital to embryo survival. There are several examples of miRNAs or their vehicles being secreted from the conceptus or taken up by maternal cells, demonstrating their promise as signals. In other cell types, the power of miRNAs as communication molecules has been shown. Moving forward, several challenges must be addressed to understand their full reach as communication molecules and to harness miRNAs as biomarkers (Figure 4).
